# The role of Allee effect in modelling post resection recurrence of glioblastoma

**DOI:** 10.1371/journal.pcbi.1005818

**Published:** 2017-11-17

**Authors:** Zoltan Neufeld, William von Witt, Dora Lakatos, Jiaming Wang, Balazs Hegedus, Andras Czirok

**Affiliations:** 1 School of Mathematics and Physics, The University of Queensland, St. Lucia, Brisbane, Queensland, Australia; 2 Department of Biological Physics, Eotvos University, Budapest, Hungary; 3 School of Gifted Young, University of Science and Technology of China, Hefei, China; 4 Department of Thoracic Surgery, Ruhrlandklinik, University Duisburg-Essen, Essen, Germany; 5 MTA-SE Molecular Oncology Research Group, Hungarian Academy of Sciences - Semmelweis University, Budapest, Hungary; 6 Department of Anatomy and Cell Biology, University of Kansas Medical Center, Kansas City, Kansas, United States of America; University of British Columbia, CANADA

## Abstract

Resection of the bulk of a tumour often cannot eliminate all cancer cells, due to their infiltration into the surrounding healthy tissue. This may lead to recurrence of the tumour at a later time. We use a reaction-diffusion equation based model of tumour growth to investigate how the invasion front is delayed by resection, and how this depends on the density and behaviour of the remaining cancer cells. We show that the delay time is highly sensitive to qualitative details of the proliferation dynamics of the cancer cell population. The typically assumed logistic type proliferation leads to unrealistic results, predicting immediate recurrence. We find that in glioblastoma cell cultures the cell proliferation rate is an increasing function of the density at small cell densities. Our analysis suggests that cooperative behaviour of cancer cells, analogous to the Allee effect in ecology, can play a critical role in determining the time until tumour recurrence.

## Author summary

Mathematical models of propagating fronts have been used to represent a wide variety of biological phenomena from action potentials in neural cells to invasive species in ecology and epidemic spreading. Here we show that when such models are used to predict the effects of external perturbations the results can be very sensitive to certain details of the local dynamics. For example, the post resection recurrence of tumour growth depends strongly on the density dependence of the proliferation of cancer cells. This suggests that targeting the cooperative behaviour of cancer cells could be an efficient strategy for delaying the recurrence of diffuse aggressive brain tumours.

## Introduction

The growth of a malignant tumour is driven by the uncontrolled proliferation of cancer cells, and their invasion into healthy tissue. While the primary therapy often involves the surgical removal of the tumour, unfortunately, the surgery often leaves a small population of cancer cells infiltrated into the surrounding tissue. After a remission period of variable duration, the surviving cancer cells can initiate the recurrence of the disease. This is a particularly serious concern for glioblastoma brain tumours characterised by a diffuse tumour boundary within a complex, heterogeneous and relatively soft brain tissue [1, 2].

A major recent retrospective MRI study has shown that 77% of glioma patients relapsed centrally within 2 cm of the original tumour mass, 18% patients relapsed more than 4 cm from the original enhancement and 4% relapsed within the contralateral hemisphere [3]. The median relapse time was 8 month for local relapses, and progressively longer for distant relapses. The median time for contralateral relapses increased almost two-fold, to 15 months.

At the macroscopic level, invasive cancers with a diffuse boundary such as glioblastoma can be described by mathematical models specifying the spatial and temporal changes in tumour cell density [4–9]. Models of tumour invasion often utilise travelling front solutions of the Fisher-Kolmogorov type reaction-diffusion equation [10–12]. Predictive quantitative models of tumour growth have been proposed as a potential tool for patient specific computational optimisation of treatment strategies such as localised radio- and combinatory chemotherapies [13–19]. In combination with diagnostic imaging, such models aim to forecast the spatial and temporal progression of the disease taking into account the heterogeneity of the tumour and the tissue environment [13, 17].

To understand the dynamics that controls the initiation of recurrent tumour growth, in this paper we investigate, using quantitative models, how surgical removal of the tumour affects its delayed recurrence. In particular, we aim to identify key parameters of tumour cell populations that determine how much the progression of cancer can be delayed by surgical resection. We show that a density dependent proliferation of the cancer cells [20], particularly at low cell densities, has a key impact on predicting the time until tumour recurrence.

## Results

### The model

We consider a population dynamics model of glioma invasion in which the population density of cancer cells within a tissue is determined by the balance of proliferation, motility and cell death. Tumour cells are known to engage in a rich variety of motility [21]. Yet, as we discuss below, available experimental data suggest that at long time scales cancer cell movement is random and well approximated as a diffusion process, similar to the behaviour observed in cell cultures [22]. Thus, tumour spreading at a tissue scale is thought to be well described by a reaction-diffusion equation of the form:

$$\begin{array}{c} \frac{\partial C}{\partial t}=\frac{\partial }{\partial x}(D\frac{\partial C}{\partial x})+Cr\left(C\right) \end{array}$$ (1)

where *C*(*x*, *t*) is the density of cancer cells at location *x* and time *t*. The diffusivity of the cells *D* characterises their random motility, and the function *r*(*C*) describes the balance of the rate of proliferation by cell division and cell death rate. In the simplest, and typically used, form of Eq (1) the environment is steady and homogeneous (*D* and *f* are independent of *x* and *t*) and the proliferation term is the logistic function

$$\begin{array}{c} r\left(C\right)=\rho (1-\frac{C}{K}). \end{array}$$ (2)

where *ρ* is the maximum population growth rate. Expression (2) assumes that, on average, the balance of proliferation and death rates of cells, *r*(*C*), decreases with the cell density and vanishes when the density reaches the carrying capacity *K*. This behaviour reflects—in a simplistic form—limitations of both biochemical resources and cell size as the cell density increases [13, 17, 23, 24].

Eq (1) with the logistic proliferation term (2) is the well known Fisher-Kolmogorov (FK) equation. The FK equation has travelling front solutions of the form *C*(*x*, *t*) = *C*_0_(*x* − *vt*) where *v* is the propagation velocity and *C*_0_ is the stationary population density profile of cancer cells, as seen in a reference system co-moving with the front [25–27]. For sufficiently localised initial conditions (e.g. with nonzero values restricted to a finite region) the asymptotic front speed is $2\sqrt{D\rho }$ and the characteristic front width is $\sqrt{D/\rho }$.

Following the surgical intervention reactive gliosis appears at the site of surgery. In the majority of the cases for a couple of months the resected area remains tissue free as evidenced by follow-up imaging [28]. As such the cell spreading into this area can substantially be delayed. Thus, in our model it is natural to represent tumour resection (or other localised primary treatments such as radiation therapy) by resetting cancer cell density *C* to zero in the region where *C* is higher than a predefined detection threshold *δ*. Back-propagation of the tumour into the area from which it was removed can be also prevented by no-flux boundary conditions imposed at the contour of the threshold density. The modified cell density profile is then used as initial condition for the same reaction-diffusion equation to generate the post-resection dynamics in the altered spatial domain. We find that our results are quite insensitive to whether or not the resected domain remains available for repopulation.

### Numerical results: Logistic growth

**Fig 1:**
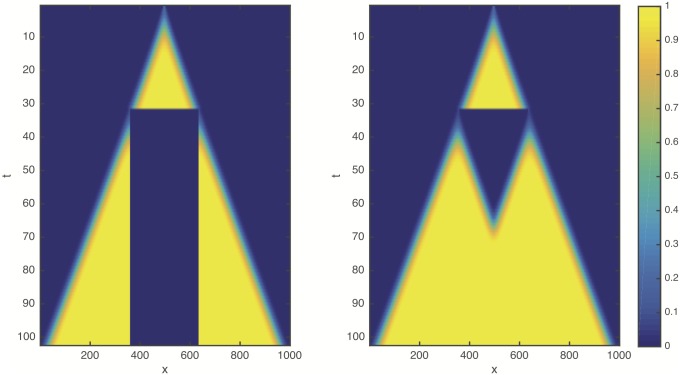
Space-time plot showing the numerical solution of the reaction-diffusion Eq (1) with the logistic growth term (2) in non-dimensional form (*ρ* = 1, *D* = 1, *K* = 1). The initial condition is localised at *x* = 500 and resection is applied at *t* = 30 with a threshold *δ* = 0.1. After the intervention the simulation is continued using the truncated cancer cell profile as initial condition. (left): The resected region is removed from the domain by imposing no-flux boundary conditions. (right): The density is set to zero within the resected region, but back-propagation remains possible.

Numerical solutions of Eq (1) with the logistic growth term (2) and resection are shown for a one dimensional system in Fig 1. Surprisingly, we find that the resection does not lead to any detectable delay of the propagation of the front: The post-resection front initiated by the truncated, low cell density tail of the cancer cell distribution coincides with the unperturbed original front (see also S1 Movie). This behaviour appears to be independent of model parameters including the resection threshold *δ*.

To explain this counterintuitive behaviour we note that in the logistic proliferation term (2) the cancer-free equilibrium state *C* = 0 corresponding to healthy tissue is linearly unstable. Therefore the FK front is a so called “pulled front” [27, 29], where the dynamics of the low cell density leading edge is not affected even by the complete removal of the population behind the front. The complete absence of a delay in front propagation, however, questions the suitability of FK equation to represent radical medical interventions, which are expected to delay the progression of cancer.

### Glioblastoma cells exhibit a weak Allee effect in culture

Since after resection the density of cancer cells is low everywhere, the recurrence of the tumour is mainly determined by the survival and proliferation of cancer cells at low cell densities. To gain a qualitative insight into the density dependence of the cancer cell proliferation rate, we performed a series of in vitro experiments.

**Fig 2:**
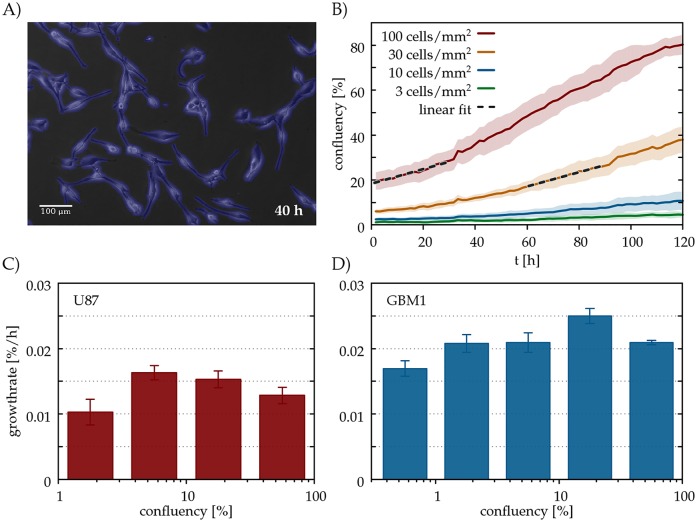
Allee effect in glioblastoma cell cultures. A: U87 glioblastoma cells in culture, detected by our segmentation algorithm (blue). (B): Confluency (area covered by cells) *A* as a function of culture time *t* in parallel cultures of the U87 cell line seeded at various densities, specified in the plot. Each line represents an average of *n* = 12 microscopic fields of the same culture. The standard error of the mean is indicated by the shaded areas. The dashed lines are linear fits over two 30 h long time periods, each starting at a confluency value of 20%. (C,D): Average growth rates *r* for various confluency values *A*, for two glioblastoma cell lines, GBM1 (C) and U87 (D). To obtain each value *r*, we pooled data from three distinct experiments, each monitoring 4 parallel culture dishes seeded at various densities. Thus, each *r* value incorporates data from 40-100 microscopic fields.

Glioblastoma cells were grown and imaged in sparse cultures for at least 4 days. Cultures were seeded at low cell densities ranging from 3 to 100 cells/mm^2^, corresponding to an area confluency (coverage) between 0.5 and 20% (see S3 and S4 Movies). We evaluated a time-lapse image sequence *s* in terms of *A*_*s*_(*t*), the total area covered by the cells as a function of time *t* (Fig 2, see Methods for further details).

The growth rate *b*_*s*_(*T*) characterising the time period *T* ≤ *t* ≤ *T* + Δ*T* was obtained as a linear fit of the corresponding *A*_*s*_(*t*) values:

$$\begin{array}{c} A_{s}\left(t\right)=b_{s}\left(T\right)t+const. \end{array}$$ (3)

We have chosen the duration of the time period as Δ*T* = 30h, sufficiently short for the linear approximation (3) to hold, and sufficiently long to detect slow changes in the area covered by cells (Fig 2a).

The density-dependent average growth rate per cell, *r*(*C*) = *f*(*C*)/*C*, was obtained as

$$\begin{array}{c} r\left(A\right)={\langle b_{s}\left(T\right)/A\left(T\right)\rangle }_{s,T:A_{s}\left(T\right)\approx A} \end{array}$$ (4)

where the 〈…〉 average was calculated over parallel cultures *s* and time intervals for which the initial *A*_*s*_(*T*) coverage was sufficiently close to *A*.

Experimental results from two glioblastoma cell lines suggest that in the low density regime the cell growth rate *r*(*C*) increases with the population density *C* while it decreases at larger densities (Fig 2). This non-monotonous behaviour is in contrast with the logistic model which assumes a monotonously decreasing growth rate. In ecology such behaviour is known as the Allee effect [30, 31], and can arise as a result of some sort of cooperative behaviour among individuals that becomes less efficient at low population density. In cultures of cancer cells such cooperative behaviour can likely arise due to autocrine growth factors, diffusive signalling molecules produced and secreted by cells that enhance growth and proliferation of other cells [32]. Mathematical and computational models of cellular mechanisms leading to the development of Allee effect in the context of tumour growth has been described in recent studies [33, 34], and properties of travelling front solutions in a model of tumour invasion with strong Allee effect was studied in [35].

### Computational model with Allee effect

Motivated by our experimental observations of non-monotonous density-dependent survival and proliferation of tumour cells, we replace the logistic growth rate (2) with a quadratic net cell proliferation rate

$$\begin{array}{c} r\left(C\right)=\rho (\frac{C}{K}+\beta )(1-\frac{C}{K}). \end{array}$$ (5)

We choose this functional form as being the simplest that describes a non-monotonous density dependent proliferation. The Allee effect can be categorised by the sign of the parameter *β* as “strong” when *β* < 0, or “weak” otherwise. In the case of strong Allee effect the spatially uniform population dynamics is bistable and there is a critical density *C*_*T*_ = −*βK* below which the growth rate is negative. For *β* > 0, the case of weak Allee effect, the cell reproduction rate increases with cell density, but it is always positive and there is no critical survival density.

The existence of a minimal density required for the survival of cancer cells would imply that the tumour can be eliminated completely if the resection threshold is sufficiently low (*δ* < −*βK*). This is, however, very rare in the case of glioblastoma [36]—suggesting that this disease exhibits a weak Allee effect: 0 ≤ *β* ≪ 1.

**Fig 3:**
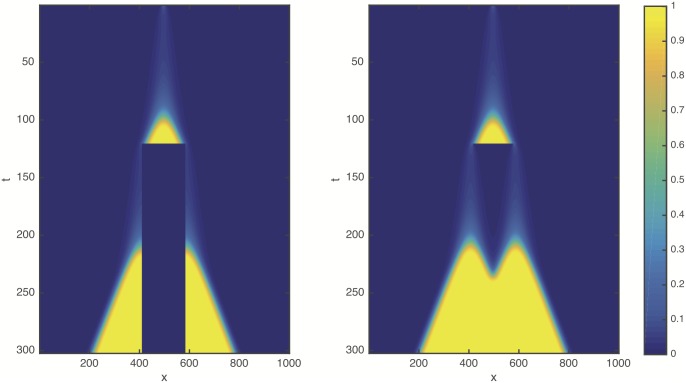
Space-time plot, similar to Fig 1, obtained with Allee effect as described by the quadratic cell proliferation function (5). *ρ* = 1, *D* = 1, *K* = 1, *β* = 0, *δ* = 0.1.

**Fig 4:**
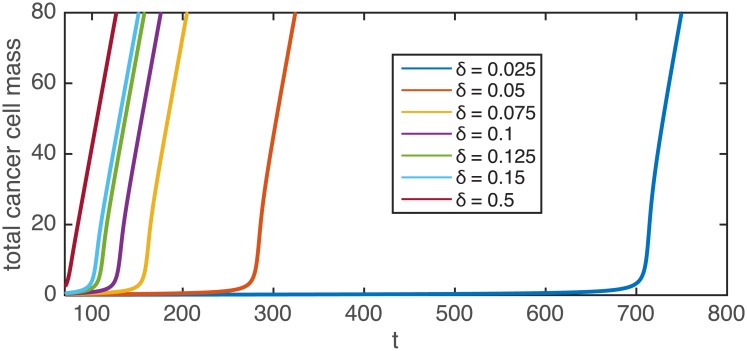
Total number of cancer cells as a function of time for different values of the resection threshold *δ* (*ρ* = 1, *D* = 1, *K* = 1, *β* = 0). A rough estimate of the time unit based on the experimentally observed in vitro proliferations rates is about 2–3 days. Note, that the case *β* = 0, shown in these simulations, gives an upper limit of the delay time, for nonzero *β* the values can be significantly lower.

We used the cell proliferation function (5) and repeated the tumour growth and resection simulations in one dimension (Fig 3). According to our expectations, in the modified model the resection can indeed substantially delay the propagation of the tumour (see also S2 Movie). Fig 4 shows the integral of cancer cell population density *C*(*x*, *t*) within the area outside the resection, for different values of the resection threshold *δ*. Note, that after resection there is a lag phase during which the total number of cancer cells is almost constant. The lag phase is followed by a sharp transition to a linear increase indicating a front moving with constant speed. From this graph we can determine the length of the remission period, *τ*, as a delay relative to the original unperturbed front. The remission period thus increases substantially as the resection threshold is reduced.

**Fig 5:**
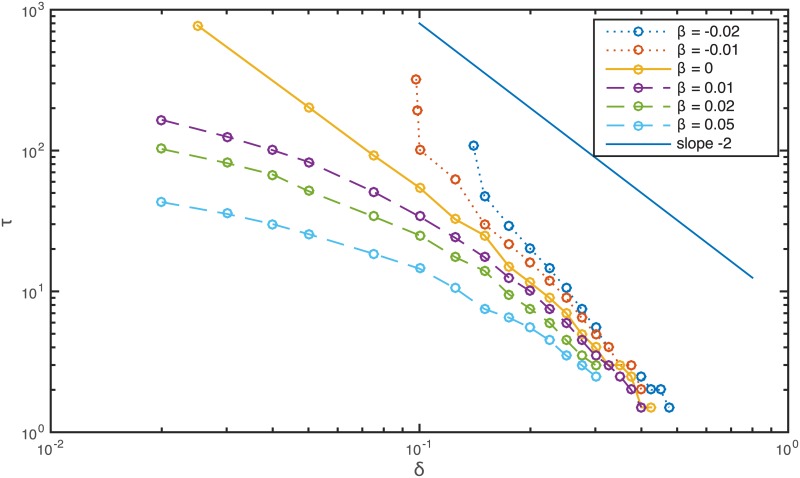
The remission time *τ* caused by resecting the tumour as a function of the resection threshold *δ* for weak and strong Allee effects.

The dependence of the remission period length *τ* on the resection threshold *δ* (Fig 5) is qualitatively different depending on the type of Allee effect considered. In the case of strong Allee effect, the delay time becomes infinite at a finite critical resection threshold. In the borderline case when *β* = 0 we find a power law behaviour where the delay is inversely proportional to the square of the resection threshold. For weak Allee effect with *β* > 0 the remission period length appears to follow a power law similar to the *β* = 0 case for larger values of the threshold *δ*, and crosses into a logarithmic function when the resection threshold is low.

### Theory

In order to explain the inverse quadratic power law dependence of the recurrence delay on the resection threshold *δ* for the case *β* = 0, we look for approximate solution of Eq (1) using the exponential tail [37] of the truncated front *δ* exp(−*ax*), where *x* > 0, $a=\sqrt{\rho /D}$, as initial condition after resection. Since we are considering the low density tail of the cancer cell distribution left from the resected tumour (*C*(*x*)/*K* ≪ 1), we can neglect the limitation of growth due to the finite carrying capacity. First we compare the relative magnitude of the diffusion and proliferation terms right after resection by substituting the initial concentration into the Eq (1)

$$\begin{array}{c} \frac{\partial C}{\partial t}\approx \delta \rho e^{-ax}+\rho \frac{\delta^{2}}{K}e^{-2ax} \end{array}$$ (6)

From this we can see that if the resection threshold is small, *δ*/*K* ≪ 1, then at this initial state the diffusion term dominates over cell proliferation. This is also visible in the numerical simulations, which show that the population density peak at the resection boundary quickly decreases at the beginning as the cancer cell population is dispersed (S2 Movie).

Although the full diffusion-proliferation equation cannot be solved explicitly, we can use this observation regarding the dominance of diffusion, and find an approximate solution valid for the initial period of time, by neglecting the proliferation term. The solution of the diffusion equation using the exponential post-resection profile as initial condition is

$$\begin{array}{c} C\left(x,t\right)=\frac{\delta }{2}[1-erf(\sqrt{\rho t}-\frac{x}{2\sqrt{Dt}})]e^{-ax+\rho t}. \end{array}$$ (7)

For large *t*, that is relevant in the small resection threshold limit (*δ* → 0), the argument of the error function is dominated by the first term, and using the asymptotic form of the complementary error function $1-erf\left(x\right)\approx \left(1/\sqrt{\pi }\right)e^{-x^{2}}/x$ we obtain the following approximation:

$$\begin{array}{c} C\left(x,t\right)\approx \frac{\delta }{2\sqrt{\pi \rho t}}e^{-\frac{x^{2}}{4Dt}}. \end{array}$$ (8)

In Eq (8) we recovered a Green’s function of the diffusion equation in which the total population size remains constant.

Now we can use this approximate solution and substitute it back into the full diffusion-proliferation equation, to compare again the relative magnitudes of the diffusion and proliferation terms.

$$\begin{array}{c} \frac{\partial C}{\partial t}\approx D\frac{\delta }{2\sqrt{\pi \rho t}}e^{-\frac{x^{2}}{4Dt}}(\frac{x^{2}}{4D^{2}t^{2}}-\frac{1}{2Dt})+\frac{D^{2}\delta^{2}}{4K\pi t}e^{-\frac{x^{2}}{4Dt}}(\frac{x^{2}}{2D^{2}t^{2}}) \end{array}$$ (9)

At the time when the contribution of cell proliferation becomes comparable to the diffusion term, the purely diffusive approximation breaks down. At this point the proliferation of cancer cells becomes non-negligible since diffusion is no longer efficient enough to disperse the cancer cell population to keep their density low at which reproduction is slow as imposed by the Allee effect. This leads to a sudden rapid increase of total cell mass initiating tumour recurrence represented by a new propagating front. Thus we can use the time needed for the proliferation term to reach the magnitude of the diffusion term as an estimate for the remission time, *τ*. Using *x* = 0 as a reference point where the cancer cell density is maximal, and balancing the two terms at *t* = *τ* we obtain

$$\begin{array}{c} \frac{1}{2\tau }\frac{\delta }{2\sqrt{\pi \rho \tau }}=\rho \frac{\delta }{2\sqrt{\pi \rho \tau }}(\frac{\delta }{2K\sqrt{\pi \rho \tau }}) \end{array}$$ (10)

which leads to

$$\begin{array}{c} \tau =\frac{1}{\pi \rho }{\left(\frac{K}{\delta }\right)}^{2}, \end{array}$$ (11)

in agreement with the numerically observed *τ*(*δ*) ∼ *δ*^−2^ for the case *β* = 0 which represents an upper limit for the finite recurrence time with weak Allee effect *β* > 0.

## Discussion

We have shown that the model of tumour invasion based on logistic cell proliferation cannot describe the delayed progression of cancer due to resection and therefore it may not describe correctly the typical outcome of clinical interventions that substantially reduce population density of tumour cells. We propose that the key element, that determines the time until tumour recurrence, is the Allee effect, which results from positive cooperative behaviour of the cancer cells.

The Allee effect at the level of a tumour cell population may reflect diverse processes at the cellular level. A number of signalling pathways that include autocrine components, such as TGFalpha/EGF/EGFR, PDGF/PDGFR, HGF/SF and CXCL12/CXCR4 ligand/receptor systems, have been identified in glioma and glioblastoma [32]. Thus, glioblastoma cells can both produce the diffusive factor and respond to its presence with the appropriate receptors that activate cell proliferation. In addition, interactions between tumour cells and the surrounding stromal cells may also depend on the concentration of growth supportive paracrine factors and thus on the local cell density [38]. Furthermore, the matrix remodelling capacity, including the deposition of fibrillary collagen that promote glioma cell invasion, is also influenced by the density of tumour cells [39]. Finally, multicellular spheroid models of tumour growth often exhibit resistance against various treatment modalities [40].

Our mathematical model that includes the Allee effect provides the following insight into the dynamics of the tumour cell population: After resection the proliferation of cancer cells is very slow therefore their distribution is mainly determined by random motility which spreads the cells into the low density regions faster than they could reproduce leading to progressively lower densities. The process is eventually halted by the density distribution of the cells near the resection boundary becoming almost uniform in space. Without a cell density gradient random cell motility cannot further reduce cell density and the slow proliferation eventually catches up and leads to the recurrence of the invasion front. In accord with this analysis, the radiologically and histologically assessed cellularity, i.e. the density of tumour cells in the tissue, is one of the most important histological prognostic factors in glioblastoma multiforme—more predictive than the total tumour burden or proliferation index of the surgical specimen [41–43]. A counterintuitive prediction of our analysis is that reduced cell motility would promote an earlier local recurrence of the disease. Experimentally, this hypothesis could be tested by comparing the migratory activity of patient derived glioma cells and the time of recurrence using a major glioblastoma cohort in order to decrease the impact of other potential confounding factors like genetic background or extent of resection.

Glioblastoma cells are known to follow extracellular matrix rich structures, myelinated tracks and tissue inhomogeneities such as blood vessels or white matter tracts (axon bundles). However, only 20-30% of glioma recurrence is non-local (occurs at a distance greater than 2 cm from the original tumor centroid) [3, 44]. Thus, remissions clearly involving directed cell migration in great excess to local diffusivity happen, but our simple model representing cell motility as a diffusive process deals with the majority of cases. While to the best of our knowledge there is no single tracking data available for glioblastoma cells in situ or in brain slice explants, in the latter experimental model cells often spread in a spatially isotropic pattern that appears to be consistent with a diffusive spreading [45, 46]. Thus, while glioma motion may be anisotropic and directed at sub-millimetre scales, the complexity of the brain tissue may result in an approximate diffusive spreading at larger scales.

The diffusion term of Eq (1) may also incorporate density-dependent effects. The random motility of cancer cells may also depend on the local cell density, hence affecting the diffusion parameter *D*. When *D* vanishes for small population densities, the diffusive FK fronts are replaced by compact fronts with a well defined boundary [47, 48]. Similarly, expansion of an adhesive tumour mass without substantial random motility would be described by an advection term. Although, such generalisations are likely to be relevant for other malignancies, the diffuse infiltrate characteristics of glioblastoma are best explained by a diffusive process with a finite *D* at vanishing densities.

Recent improvements in imaging technology offer the promise of treatments specifically optimised both for the individual patients and tumours at the specific locations. We demonstrated that predictive models of tumour progression, necessary to evaluate and design such treatments, must include the Allee effect of tumour cell population dynamics. In this paper we considered a highly simplified one-dimensional model. In reality the strongly non-uniform tissue environment distorts the shape of the tumour and influences the cell’s ability to migrate. Although this will not modify the main qualitative observations regarding the relationship between tumour recurrence time and the Allee effect, such inhomogeneities and tissue anisotropies need to be taken into account when optimising treatment modalities in a patient specific manner. While surgery always aims to remove most of the tumour cells, our results indicate that interfering with autocrine feedback regulation of growth control at low cell densities may effectively prolong remission after surgery. As areas with maximal cell densities (and not the total tumour burden) determine remission time, radiotherapy optimisation must also critically depend on the Allee effect.

## Methods

### Cell culture

Two human glioblastoma cell lines (U87 and GBM1) were investigated in this study. U87 is a standard cell line from American Type Culture Collection (ATCC, HTB-14), GBM1 was established from a giant cell variant of glioblastoma multiforme at the National Institute of Neurosurgery in Budapest, Hungary as described previously [22]. Cell lines were maintained and studied in Dulbecco’s Modified Eagle Medium (DMEM, Lonza) containing L-glutamine, supplemented with 10% fetal bovine serum (Invitrogen) and penicillin-streptomycin-amphotericin B (Lonza). Cells were grown in non-precoated culture dishes at 37°C in a humidified, 5% CO_2_, 95% air atmosphere. Confluent cultures were washed twice with PBS (Invitrogen) and incubated with trypsin-EDTA (Sigma) to obtain cell suspensions. Cells were seeded in low densities (3, 10, 30 cells/mm2) into 35 mm Petri dishes (Greiner).

### Time lapse imaging

Time-lapse recordings of the cell cultures were performed on a computer-controlled Leica DM IRB inverted microscope equipped with a Marzhauser SCAN-IM powered stage and a 10x N-PLAN objective with 0.25 numerical aperture and 17.6 mm working distance. The microscope was coupled to an Olympus DP70 colour CCD camera. Cell cultures were kept in a stage-top mini incubator at 37°C in humidified 5% CO_2_ atmosphere. Phase contrast images were collected every 10 minutes from each microscopic field for durations up to 3-4 days.

### Image analysis

Recorded phase-contrast images were analysed by segmentation and particle image velocimetry (PIV) algorithms implemented in Octave and Python. To detect cell occupied area a global threshold was applied to the local standard deviation of intensity on each image [49]. The code used for segmentation and confluency calculation are available at http://github.com/aczirok/cellconfluency.

## Supporting information

S1 Movie Movie showing the front propagation before and after resection using the logistic proliferation term. Blue curve is the original unperturbed front and Red dashed curve is the post-resection front. Note, that in this case the post-resection front coincides with the unperturbed one, i.e. the front propagates without any delay. The parameters are *D* = 1, *ρ* = 1, *K* = 1, the resection time is *t*_*s*_ = 20 and resection threshold is *δ* = 0.1.
(AVI)

S2 Movie Movie. Same as S1 Movie. except that the proliferation term is replaced with the cubic function including the Allee effect (*β* = 0). In this case the resection is followed by a latent remission period and the recurrent front is substantially delayed relative to the original front. The parameters are *D* = 1, *ρ* = 1, *K* = 1, the resection time is *t*_*s*_ = 70 and the resection threshold is *δ* = 0.1.
(AVI)

S3 Movie Movie. Segmented time-lapse phase-contrast image sequence of the U87 cell line.
(MP4)

S4 Movie Movie. Segmented time-lapse phase-contrast image sequence of the GBM1 cell line.
(MP4)
